# Rapid implementation of remote digital primary care in Stockholm and implications for further system-wide implementation: practitioner’s and manager’s experience of the Always Open mobile application

**DOI:** 10.1080/02813432.2023.2229387

**Published:** 2023-07-20

**Authors:** Karin Solberg Carlsson, John Øvretveit, Mikael Ohrling

**Affiliations:** a Department of Learning, Informatics, Management and Ethics, Medical Management Centre, Karolinska Institutet, Stockholm, Sweden; b Stockholm Health Care Services, Region Stockholm, Sweden

**Keywords:** Covid-19 pandemic, primary care, telemedicine, management, NASSS framework

## Abstract

**Objective:**

To contribute actionable knowledge how to increase appropriate use of digital technologies in primary care by understanding clinical managers experiences with a digital connection system, Always Open, during the COVID-19 pandemic.

**Design and subjects:**

The overall design was a qualitative study with directed content analysis method. Data were collected from documents and focus group (n = 12) interviews with clinical managers (n = 99) of primary care. The seven domains of the Non-adoption, Abandonment, and challenges to the Scale-up, Spread and Sustainability (NASSS) framework was used to understand the implementation process, as described by the clinical managers.

**Results:**

Focus group participants reported that their units made their own local decisions to make more use of the technology provided by the health system. Most participants considered that the technology was ready to use, despite some limitations, that included individual clinician’s and patient preferences, and how ready their unit was for making changes to practice and organization. Some raised concerns about how standardizing some aspects possibly conflicted with the decentralized management model of the organization. The overall experience was reported to be positive, with an intention to sustain the achievements.

**Conclusion:**

Focus group interviews found that clinical unit managers reported that they and their staff were positive about the digital technology system for remote care. For the future, they wanted changes to be made at different levels of the health system to better combine digital and physical care. Possibilities to use digital technology to integrate primary and hospital health care were identified.

## Key points

Rapid adoption of Always Open as a digital connection mHealth system for remote digital care was observed during the pandemic without a structured implementation program.

Many factors could explain the rapid adoption and the intention to sustain the use of Always Open such as professional ethics, financial reimbursement, patient needs and support.

Standardization needs to be balanced with flexibility to ensure that staff can decide what is appropriate for patients.

The general experience was positive and a continual high use of Always Open was reported in units that adopted the system.

The decentralized management model may have contributed to the capacity to provide flexible services suited to local needs.

## Introduction

Most health organizations plan to make more use of all types of digital technologies as a strategy to address challenges in patient demands, access, efficiency, and staffing [1,2]. For clinical care, one constraint in the past has been clinician’s low adoption of digital healthcare in their everyday practice, even when they have been provided with well-designed systems, training, and support [3,4]. For better or worse, the early days of the Covid-19 pandemic made digital connections the only option for many patients and clinicians. At the peak of the first wave of Covid-19 a 16-fold increase in the total weekly patient video consults to public primary care centers in Stockholm via an mHealth application (app) was observed compared to the same period one year before. However, this change was from a low number of 2000 for the population of 2.4 million.

What were clinician’s experiences, and can we learn lessons from this to enable the adoption of digital technologies by clinical units? This was one of the questions addressed by our study of clinician managers’ experiences using one clinical digital connection system (CDCS), which was a smart phone application called Always Open (AO), in their public healthcare delivery services in the first eighteen months of the pandemic. This report first presents the previous research on this subject that we considered to help to identify the knowledge needed and methods that we might use in the study and then describes the study setting and methods before presenting the findings.

### Previous research

For many people digital contact with health care was a safer method in the early months of the COVID-19 pandemic, made care accessible and reduced delay to gaining health care. Overall, research showed that digital visits could usefully enable contact between patients and health care providers and substitute for physical visits in some situations [5]. Many of the digital mHealth solutions taken up had been in use for some long time before the pandemic, but had not been adopted so widely [6–8]. Questions were raised as to whether the early rapid and extensive take up would be sustained, but there have been few studies to answer these questions [9].

There are empirical studies into primary care video consultations before and during the early part the pandemic. These include studies of patient experiences in Sweden [10–12] and studies of physician’s experiences (Sweden [13,14]; New Zealand: [15]), and a study of patient’s and physician’s experiences in Scotland before the pandemic [16], and in England in the early part of the pandemic [17]. A review one year before the pandemic, found limited evidence of healthcare professionals’ work satisfaction when using video consultations, and that the evidence base was “equivocal” [18]. One Swedish interview study in Swedish primary health care, guided by the Job Demand-Control-Support model, found that physicians perceived working with digital consultation as flexible with high autonomy and reasonably low demands. However, most thought that if medical skills and abilities are to be maintained, full time work with remote digital care would not be possible [13].

There is research that considers on-line care services for people experiencing mental health challenges, and for other specific patient groups such as those diagnosed with cancer, diabetes, and heart disease, but most before 2020 [19]. We could not find any that report evidence of the experience of personnel in primary and community healthcare during the pandemic who used digital technologies for more than 12 months and covering the different later phases of the pandemic such as those associated with the delta and omicron variants of the virus.

As regards clinical digital connection systems (CDCS) enabling provider and patients to make secure connections, there are several commentaries and blog publications. Our search and the two most recent reviews in 2021 did not find any empirical studies of provider’s experiences with CDCS or patient portals in Sweden [20,21]. No studies of clinician level manger’s experiences with CDCS or patient portals were found in the wider search. There are some commentaries about ordinary telephone consultations during the pandemic which appear to have been the preferred method by many practitioners, especially for talking to vulnerable patients [22,23]. The research suggests that provider use and experience with CDCS is largely influenced by characteristics of the system, the implementation strategy, and factors of the context.

Studies about the use of remote digital technology in response to the pandemic have mainly reported on a specific type of technology, patient need, or the health care system targets addressed. The scientific contribution of the research is limited regarding implementation strategies. In a recent review by James et al [21], findings from 13 papers were synthesized. They concluded that there is an urgent need for more evidence to “support global efforts and match enthusiasm for extending use.” In their review Golinelli et al., [5] concluded that national health systems, “have been proved to be particularly resistant to the digital transition in recent years” and proposed the need to keep track of models driven by the pandemic to better understand the implementation of digital solutions.

Overall, the search found patient and provider experience depended in part on the particular technology or CDCS used to connect them. There was limited evidence about these subjects later in the pandemic when people had more experience and had returned to more face-to-face physical visits. In addition, there is little evidence of implementation strategies for rapidly scaling-up remote digital services such as video consultations in a large public primary and community service delivery organization and no evidence of the experiences of service delivery unit managers. It is possible that adoption of remote digital patient clinical consults by clinicians in primary care could be informed by understanding more about their experiences and those of their unit managers with this patient contact method during the pandemic.

Our review of previous research did not find any theoretical frameworks for understanding service delivery level clinical connection systems. We found one general framework which had been used in over 20 studies of digital technology implementation and use in healthcare, and which could provide guidance for research into factors that might explain uptake and utlisation levels. This was the NASSS framework (Non-adoption, abandonment and challenges to Scale-up, Spread and Sustainability) [24].

The NASSS framework was developed to “help predict and evaluate the success of a technology-supported health or social care program” [24]. We chose this framework because it was suited to the study objectives and allowed an analysis of the difference components of the system and their interaction. In addition, it had been developed in a public health care system and systematically pilot tested in six healthcare organizations and has been used successfully in a number of studies [24]. Two studies have applied NASSS to assess digital technologies similar to the technology considered in our study. The first study applied NASSS in a review of video consulting in healthcare [21]. The second study used the NASSS to develop a caregiver portal [25]. We used these as examples to check our interpretation of the seven domains against how these studies had classified their data in different domains.”

### Description of the Stockholm clinical data connection system, implementation, and context

Digital access and remote digital services had been introduced two years prior to the onset of the pandemic, but not taken up by many patients or providers. The mHealth service was a smart phone application (app) Always Open (AO) that gave access to online services for the public and for primary healthcare physicians and other public services in Region Stockholm, Sweden. The application gives the provider or patient (user) access to a digital operating system that provides the following secure connection functions to patients for providers: video consults, telephone, chat, and SMS text and provides a booking function and other services for patients [26,27]. AO is part of a larger clinical digital connection system (CDCS) that collects and transfers information between clinicians and between them and patients, as well as linking to other data bases.

Before the pandemic, Region Stockholm (RS) had contracted four on-line only doctor services to provide these services to Stockholm residents at no cost to the resident. This was continued during the pandemic, but later RS contracts required that for any on-line doctor service to be reimbursed the service must also offer the patient a choice of a physical visit in the region [28]. This led a few digital-only services who wanted to continue these contracts to open new physical primary care units in the Stockholm area. At the time of the focus group interviews in this study, this requirement was not known to most members of the groups.

Region Stockholm has broad strategies to develop digital health, but no significant implementation strategies in the primary care units [28,29]. With the rapid onset of the Covid-19 pandemic in Stockholm, the scale-up of digital care occurred by patients and providers using the Always Open mobile application but without a formal management directive to do so, or a structured or planned implementation process.

### Trends in number of patient video consultations with public primary healthcare centers

There were large differences between primary health care units in the number of patient video consultations they carried out, and between other community health care units. In Stockholm, there was no systematic implementation, and local units independently explored how the technology could be used for their different patients and uses. A central support function was available to these units to help with questions and issued that they raised [26–28].

### Research objectives

Given the limitations of the knowledge about the use and uptake of digital clinical connection methods in primary care that we found, we defined the research objectives as to provide scientific knowledge that, 1) describes and explains how a digital application for virtual care was taken up by providers in primary care during the first 18 months of the Covid-19 pandemic, without an explicit and supported implementation strategy, 2) can assist health care systems that are implementing or sustaining the use of similar clinical digital connection systems.

There are no already-developed theoretical frameworks specifically to investigate this subject. We assumed that the system-wide introduction and implementation of an innovation such as AO might be affected by factors outside the control of the developer of the innovation. Therefore, we chose the NASSS framework to guide data analysis because it covers many aspects of components in a health care system. In addition, we also sought to assess the usability of the framework for a large-scale technology that is not specifically designed for a limited medical purpose.

## Methods

### Study setting

Stockholm County Health Care Services (Swedish abbreviation, “SLSO”) is a tax financed public healthcare delivery organization that provides community-based health care services, including primary care, for 2.4 million inhabitants [30]. The organization is separate from public acute care hospitals, and includes 68 primary health care centers, employing medical practitioners, nurses, administrators, as well as other community-based health care services such as psychiatric care, geriatric care, and habilitation services. Private providers are also contracted by the Region Stockholm Government (RS) for publicly tax-funded primary and community health care in this mixed public and private service delivery healthcare system [31]. Clinical managers, also called service delivery unit-managers, in SLSO have a large degree of delegated managerial authority that matches their accountability for budgets, for managing employee performance and satisfaction, and for actions taken by the unit. Otherwise, the organization is a typical line-management public bureaucracy structure with value- and trust-based governance [32–34].

### Study design

The design chosen was a qualitative study, using a directed content analysis [35]. To collect the providers experiences about remote digital healthcare and the system that connected them to patients, we chose a focus group interview method as the most appropriate for the research objectives [36]. This method allowed flexibility to explore issues raised in the group, and also contributed to their shared learning from their colleagues’ experiences. Other data was collected from published documents. The design followed the COREQ checklist for qualitative research [37].

Table 1 summarises the seven domains of NASSS that we used to guide data collection and analysis, and which cover, the condition, the technology, the value proposition, the adopters, the organisation(s), the wider system and embedding and adaptation over time.

**Table 1.** Main domains of the NASSS-framework. We aimed to produce a theory-informed, analysis of our findings, and consider all seven domains of the NASSS framework, in order better to understand the constraints and facilitators affecting implementation described by the focus group participants.

NASSS domain	Description
**1. The condition**	Describes the nature of the illness or condition, and relevant co-morbidities and socio-cultural factors
**2. The technology**	Describes the technology’s key features, the knowledge it brings, the knowledge required to use the technology, the technology supply chain, and who owns the intellectual property to the technology
**3. The value proposition**	Describes the developer’s business case (supply-side) and the technology’s desirability, efficacy, safety, and cost-effectiveness to users (demand-side)
**4. The adopters**	Describes implied changes required in staff roles, practices and identities, expectations on patients and/or immediate carers, and assumptions about extended network of lay carers
**5. The organisation**	Describes the organisation’s capacity to innovate, its readiness for technology-supported change, decision making processes for adoption and funding, changes needed to team interactions and routines, and work required for implementation
**6. The wider system**	Describes political context related to the implementation, regulatory context, position of professional bodies, socio-cultural context, and the extent of inter-organisational networking
**7. Embedding and adaptation over time**	Describes the scope for adapting over time and the organisation’s resilience to handling critical events and adapting to unforseen eventualities

### Study participants

To minimize the burden on personnel at a time of high workload and staff sickness and to get a wide perspective, we chose the clinical managers of the service delivery units. They also worked as clinicians in their unit and had a detailed understanding of the issues involved for all clinical and administrative personnel in their unit. In total, 99 clinical managers (13 men, 86 women, Appendix 1). The participants were grouped according to their specific service within the organization: primary health care, psychiatry, geriatrics, rehabilitation, out-patient somatic specialist health care, and advanced care in the home. All the different services of the SLSO organization were represented. The clinical managers had a background as doctors, nurses, physiotherapists, or psychologists, and various levels of managerial skills and experience.

### Data collection

Data about experiences with the system was collected in twelve focus group interviews, conducted between 15th September and 25th November 2021 about participants’ experiences with the clinical digital connection system between March 2020 and October 2021. One researcher (KSC), experienced in qualitative methods and without any established relationship with the participants, conducted all focus group interviews using a semi-structured interview guide with four open-ended questions (Appendix 2). The study took place at different facilities in Stockholm, for eight groups physically, and for four groups digitally, due to a later increase in infection levels. Each focus group interview lasted one hour involving two to sixteen persons participated in each. The researcher took detailed notes of what was said by the participants and discussions were recorded to review certain details afterwards.

### Data analysis

The text from the interviews was analyzed using directed content analysis of data from the 12 focus group interviews and the published documents following Hsieh and Shannon [35]. The codebook used was based on the NASSS framework (Appendix 3). The researcher (first author, KSC) performed the coding. To establish trustworthiness co-authors (MO, JO) separately verified the analysis, and all authors agreed upon the final qualitative analysis. Information from the interviews was checked with corresponding information found in documents about Always Open (AO) and digitalizing patient care. Validation was achieved by presenting the findings to divisional managers and the management team and documenting any corrections or misunderstandings as well as from published documents about AO [36]. To illustrate results for the reader, quotes that exemplify certain findings are presented in the results section below, drawn from the researcher’s notes from the focus group interviews.

### Ethical approval

The study received ethical approval from the Swedish Ethical Review Authority, reference number Dnr 2020–01521. Informed consent was obtained from all clinical managers to participate.

## Results

### Focus group findings

The analysis of the focus group data into the seven domains of the framework resulted in 290 codes (Table 2). The dataset generated during this study with sub-categories and sub-sub-categories is presented in Table 3.

**Table 2.** Summary of the number of codes assigned to each NASSS domain

NASSS domain	Number of codes
Domain 1: The condition or illness	2
Domain 2: The technology	53
Domain 3: The value proposition	80
Domain 4: The adopter system	51
Domain 5: The organization	53
Domain 6: The wider context	40
Domain 7: Embedding and adaptation over time	6
OTHER	5
**TOTAL**	**290**

**Table 3.** Main NASSS categories and sub-categories derived from analysis of findings

Main categories (based on NASSS domains)	Sub-categories (derived from interview data sorted under specific domain)
Domain 1: The condition	The innovation is not closely connected to any specific condition or specific patient group
	Regardless of the patient's condition, every patient's need ought to dictate how care for him or her is delivered
Domain 2: The technology	The technology works but is perceived as clumsy and needs to improve in functionality
	Always Open's content ought to generate added knowledge
	Always Open experiences tough competition
	Always Open will require continuous maintenance of the required software and hardware
	Further development of Always Open's role in Region Stockholm is hindered because it is not available from a computer
	There is a perceived risk that people will give up on the app
Domain 3: The value proposition	Always Open ought to contribute to accessibility and continuity
	Always Open has resulted in new ways of delivering care
	Always Open provides staff with more detailed patient information
	Always Open has made care visits more accessible to patients
	Digital visits save time and energy for patients
	Digital visits can increase the quality of specific visits
	Digital group visits require more resources than physical ones
	Always Open has affected daily work in a positive way
	Always Open has the possibility to change SLSO's role in Region Stockholm
Domain 4: The adopters	Employees experience a lack of continuity and structure when working with and training in Always Open
	Many patients have adapted well toward a new type of care visits, but health care needs to be attentive to each patient's preferred type visit
Domain 5: The organisation	The organisation's capacity to change
	The organisation's readiness for the technology
	The extent of changes to work routines related to Always Open
Domain 6: The wider system	Regulations may hinder further development of digital care
	Micromanaged reimbursement models may hinder further development of digital care
	Society is stuck on the idea of traditional primary care facilities
	The region's acute hospitals do not have enough knowledge about Always Open and that they can use the app at their own facility
Domain 7: Embedding and adaptation over time	Several improvements were made to the app during the first year of the pandemic

### Domain 1: The condition or illness

The focus group participants described Always Open (AO) as an innovation, which made it possible to help patients with many different types of illnesses or conditions, and during different phases of their illness. Related to this, the participants emphasized the importance of letting every patient’s need, regardless of illness or condition, guide how their care was delivered, including preventative visits and letting the patient choose between a digital or physical visits.

### Domain 2: The technology

All focus groups discussed different functions that AO performed for users, including those that they wanted in the future. In general, the groups said that AO worked better than most other communication tools that they had used in their work, and several expressed a wish to move all of the organization’s digital services or services into the app, thus allowing access to all through this one platform. This included the patients’ electronic health record (EHR). However, some groups said that the app’s interface design was “clumsy”, old-fashioned, and difficult to use. Some mentioned a risk that staff and patients may give up using the app if it is too difficult to work with. To practice how certain functions worked before seeing patients, a suggestion made in one group was to create for providers a virtual patient that they could use for a test before the consultation.

*”We want to be able to try out functions in a test environment rather than testing in actual patient-facing situations, in order to check how it works. We need a safe test environment with a pretend patient”.*

This same group, and some others, also emphasized the importance of using the chat function to improve triage and auto-generate referrals for blood sampling and follow-up. One group suggested using the app to recruit patients for research, especially for clinic-based research by staff.

*”We want to be able to recruit patients. “Are you interested in contributing to research?” ought to be one of our offers”.*

Competition from numerous private remote digital care providers was identified as a possible threat to wider implementation and sustainment of Always Open, unless the app was continually developed, simplified and made easier to use by those who did not use it. However, a number of groups observed that private online providers had successfully entered the remote digital health care market in earlier years and had driven SLSO to develop its own app.

*”Had [competitors] and others not existed, we wouldn’t be sitting here discussing digitalization today”.*

### Domain 3: The value proposition

Many groups observed how AO added to the value of their service by enhancing” accessibility and continuity” and also described other factors that could add value for both patients and staff. Several group participants mentioned that their patients can conduct certain tasks online on their own or take part in real-time learning sessions via the app, in addition to certain physical examinations and treatments. Others mentioned examples of ways in which video consults increased the quality of the visit. One mentioned the value to providers of seeing a patient in their natural surroundings, such as a child with a neuropsychiatric disorder having breakfast. Some reported that younger patients often feel more relaxed talking via video rather than face to face because they are more used to this type of communication. Others mentioned that patients with immunosuppressed conditions, fatigue, or severe bowel problems, get “more out of every hour” with their clinician when they are not stressed with the discomfort of travel.

However, although the quality of visits for some patients may be higher, the participants of some groups observed that more resources are sometimes required to provide digital visits than for physical ones. Digital group visits were said to require an extra person to deal with technical issues that may arise during a learning session.

*”You have to have two staff members during the digital group meetings. The first one has to be able to focus on the patient work while the second one functions as a “digital receptionist” in order to be able to help the patients with technical issues in case they accidentally drop out of the meeting”.*

The functionality that allowed patients to book digital visits was said by some participants to have the potential of reducing the number of phone calls for appointment booking, as well as making it possible for patients to book outside of opening hours. Some participants reported that digital visits also had resulted in fewer late cancellations, and increased accessibility to language translators. Also, that it made it easier for personnel to work at home and increased flexibility of working, all of which increased staff work satisfaction. Several groups said that AO could possibly become the region’s main patient gateway for those seeking care and a coordinated single-entrance because so many inhabitants had downloaded the app.

### Domain 4: The adopter system

As reported above in the description of AO and the CDCS, there was no systematic implementation strategy to enable take up and use of AO, although online training and information was available to providers later in 2020. The groups discussed factors related to both patients and unit staff that may influence the implementation and success of AO. Many participants said that their staff had not received structured learning to work with the app. As the pandemic developed, rapid changes were made to the app. which meant that staff were “left on their own” to learn about ways to deliver care via digital tools. All groups expressed a wish to learn from these experiences, and to standardize certain procedures in digital care pathways involving different units across the region to utilize the new technology in an optimal way. Some specifically wished to learn how to improve their patient-education skills for digital visits.

As regards adoption by patients, most group participants took the view that many patients had adapted well to the digital way of seeking and receiving care. Several mentioned that patients often conduct care-related tasks from home, such as blood pressure monitoring, filling out forms and assessment scales, booking appointments, and seemed to take greater responsibility for their own care. Participants also mentioned the importance of staff trusting their patients to perform these tasks correctly and to report back to their clinician if they had any problems.

*”Almost all patients are positive towards digitalization of care visits. They mention that some improvements are needed but basically nobody wants to return to the old ways of doing things”.*

Clinicians’ preferences differed as regards how much of their time working with video consults was too much. One group discussed how large a percentage of clinicians’ time on video calls was “reasonable - perhaps 20%”, but that 6% was “perhaps too little”. Some took the view that “high” levels of video consults that some clinicians had carried out in the early months of the pandemic was not sustainable for them. Some wanted flexibility to choose and to share the video visit demand across the unit according to clinician preferences.

### Domain 5: The organization

The groups discussed several factors within SLSO that may influence how AO is implemented and utilized. Many participants indicated that SLSO is capable of rapid change and improvement. The most mentioned example was the fast transition from traditional physical visits to digital visits by the service delivery units when the pandemic started. Others were that SLSO central support unit developed video education quickly for individual patients as well as groups.

However, many participants said that the knowledge of some managers and personnel about their own organization (SLSO) was too low, not knowing how a small change might affect other services within the same organisation. Some participants did not know about the SLSO out-of-hours online-only doctor care service for all patients, called Family Physician Online (in Swedish HLM Online), accessible only via AO.

*“I hardly knew HLM Online existed and I have always referred my patients to the local emergency room or to one of our competing online practitioners. From now on we’ll try to refer more patients to HLM Online”.*

Non-standardized pathways for seeking digital care was said to hinder the SLSO’s value proposition of being able provide accessibility and continuity of care in an integrated health system. Some participants also commented that the organization had yet to decide how to triage and refer patients effectively via the app and using their HLM Online unit. Several groups discussed, without reaching consensus, options such as triaging all patients using HLM Online, or triaging at the patient’s care facility of choice, or both.

*”How can the (physical) care facilities increase their collaboration with HLM Online? What should the patient’s way-in look like?”*

SLSO’s technological readiness for AO was generally described in the groups as high, with adequate internet connectivity and hardware. Participants described a high willingness of unit personnel to work with the technology but stressed the importance of organized practical training, as well as practical manuals, to minimize problems.

*”There ought to be good central information on how to carry out digitalized care. All knowledge regarding patient accessibility exists, for example for patients with impaired vision, but it ought to be gathered in one place and in an easily accessible way”.*

Participants described several changes to work routines that were, or may be, required to increase the number of digital visits. Some managers expressed concerns that the digitalized way of delivering and coordinating care might increase costs for their unit. Some were concerned about a possible growing case mix of the region’s more complex or “more difficult patients” when offering increased continuity via AO, and that private digital-only providers could refuse to serve at the time the groups were held.

Two groups described new management challenges with more staff working remote. Unit managers are responsible for working environment, but when staff work from home there may be ergonomic issues and there is some indication that personnel work longer hours than scheduled. Some also mentioned that creative teamwork is more difficult to conduct remotely. Several managers described digitalization as a continuing process and stressed the importance of allowing themselves time every week to keep developing the systems and practices at their unit.

### Domain 6: The wider system

All groups described factors outside of the organization at the regional or national level that helped or hindered their implementation of digital services in their unit and the functionality of AO. Several groups took the view that AO could and should become Region Stockholm’s main patient portal for coordinating care and referrals. However, such further development is hindered by the legal requirement that specifies that, for AO to become the main contact point in the region, it needed to be accessible by patients via computer which it currently is not. Another hindering regulation is the European General Data Protection Regulations [38] which restricts use and exchange of sensitive personal information. This affects how digital group sessions are conducted, either with multiple patients or with multiple clinicians, and affects the choice of communication platform used for certain types of conversations.

*”We are not allowed to use Teams (Microsoft) for any work regarding patients but sometimes we have done so anyway, if we have decided beforehand not to mention patients’ names, in order to be able to show film clips or program software”.*

*”Patient safety issues (regarding digitalized care) have been driven too hard and it often hinders digitalized care to be delivered. It makes it difficult to conduct network group meetings regarding children, sign language lessons for parents, sharing teaching material, and film clips”.*

Several groups mentioned that accessible and equitable care for all may be hindered because AO requires the person to use digital identification (commonly a bank ID application that verifies their identity), to connect to make appointments or to take part in remote digital care visits. Some group participants described having to use “work-arounds” for people without a digital ID.

*”In order to be able to conduct distance contacts with patients registered with us who don’t have digital IDs, but who have a smart phone, we in the unit contact the patient and make the connection at the time of the appointment.”*

Several groups described that their reimbursement for visits favor physical over remote digital visits. They described that the regional purchaser organization has made detailed decisions regarding reimbursed services.

*”The purchaser organization has micro-managed which activities should be reimbursed and how, which has resulted in, for example, new visits to speech therapists need to be delivered physically and follow-up visits can be delivered digitally, even though it is better to do it the other way around”.*

Some suggested that increasing the capitation part of the reimbursement scheme could be a method to achieve a more channel-neutral reimbursement for services delivered, be they physical or digital visits, or via telephone. They said it would be important that the reimbursement be closely related to resource utilization.

Some groups described both patients and society at large as “stuck” on the traditional idea of physically visiting one’s family physician at the local health care unit regarding minor conditions, rather than booking a digital visit and or using the region’s online-only doctor service (HLM online).

*”Some ask:” But aren’t I going behind my care facility’s back?” [to use HLM online]. Patients are not aware that their physician can read HLM Online’s patient records. This is something that the clinicians need to inform their patients about.”*

A last wider system issue raised was competition with the private on-line only doctor services that the region had contracted to provide services before and during the pandemic. Positive aspects raised by members of some focus groups was the competition had driven SLSO to develop its own public on-line services (HLM online) and had reduced workload for the public service units when patients used the private on-line service, mostly for simple medical problems. However, members of some focus groups felt that there was unfair competition. They reported that on-line services advertised their services extensively, but public services were restricted from public advertising in the same way. Also, that the method used by this online service did not make clear to patients that they might mistakenly transfer their registration from their primary health care unit to the online doctor, and hence the unit loses the capitation funding and causes confusion for patients if they do not understand. A third consideration was that public services could be left with more complex patients needing more time, for which the services were not fully reimbursed.

*"If we compete with continuity, we get heavier patients, but we need to lighten up with a little lighter patients so that the work environment is reasonable.”*

### Domain 7: Embedding and adaptation over time

As regards adaptation of the CDCS and Always Open system, most groups commented that the SLSO informatics team and others make changes during the first 18 months of the pandemic that had increased its functions and usability for patients and providers, such as the possibility to conduct group visits. Many also described changes to patients’ traditional care processes by combining digital and physical visits as part of the treatment plan.

*”You can do a mix – digiphysically”.*

*”You can work both ways and you should work both ways. Not one or the other. We cannot box ourselves into certain systems. We need to remember that we work with fellow human beings who have lives outside being a patient, and that we ought to help each other”.*

## Discussion

### Different and new knowledge

Our search found limited research or evidence-based guidelines that could help primary care clinicians and patients to decide for which patient conditions a video or other digital consult was appropriate, and when a physical visit was necessary [39]. All services in our study reported that the AO application connecting patients and providers was useful regardless of the patients’ condition. Also, we could not find evidence that could help the personnel to change daily work routines to make more use of video consults and other digital technologies, or to re-organize systems to integrate digital and physical care optimally and to include future likely technologies that would save time and provide better services to patients. In our study the need for changes to work routines was highlighted in all groups.

Coming out of the pandemic, it was difficult for the participants to determine what level of remote digital versus physical visits was appropriate. These observations are consistent with reports about rapid scale-up and a substantial shift to remote digital health in several health care systems [39–42]. What is the “new normal”? During the most intense months, understandably patients and clinicians would book video visits to a very large degree, but further research is needed to explore developments of various “digiphysical” care paths. In NHS, Greenhalgh et al found that video consultations became less widely used after the pandemic because many issues could just as easily be solved over telephone [43]. Kaiser Permanente in California found that video visits appear to generate more clinical actions than telephone visits in the eight diagnoses included in their study, particularly in clinical areas where the clinician required visual information to make an informed decision [44]. Tasneem et al present similar findings to our study, namely that patients saw benefits regarding increased access to care and reduced discomfort from traveling. They support a mix of video consultations and physical examinations for patients with cancer [45]. In addition to that, Viers et al found that video visits reduced travel costs for patients compared to physical visits and Yan et al suggest that users’ satisfaction with the overall quality of the video visits is the strongest predictor for its continued use [46,47]. We anticipate finding similar results to these in our future studies of AO.

As regards digital equity, some focus group members reported that the CDCS increased equity of access for some patient groups who were less able to make a physical visit. Of note, however, was that none commented that the CDCS made it more difficult for patients who could not use AO to access or use healthcare. Other research has drawn attention to factors that can disadvantage or exclude patients from remote digital healthcare, such as literacy, education, access to a digital device or the internet [48,49], age and ethnicity. Due to this, Huang et al emphasize the importance of keeping a telephone option for distance visits open [50]. This may further support our participants’ suggestion for channel neutral reimbursement.

After 18 months of the pandemic, and when most patients were making physical visits, most focus group members reported not only to continue making use of the system, but also wanting to be actively involved in developing it. All groups expressed a wish to learn from the pandemic experiences, and some proposed standardizing procedures for digital care patient pathways involving different units across the region to make more use of the benefits of the new technology and avoid the provider problems with multiple patient access. Some expressed a wish to learn how to improve their patient-education skills for remote digital visits. These findings contribute some beginning evidence to answer questions of whether the level of use would be sustained after the early months of the pandemic when there are higher levels of virus immunity in the population [5,51]. Some other studies found that video consulting was abandoned or reduced because the system adopted was not user-friendly, or because of the centrality of multidisciplinary teams in diagnosis and care [52].

### Implications for practice and research

The findings complement those of a pre-pandemic Swedish study of patient perceptions, a post pandemic study of work tasks in primary care, and telemedicine studies of providers in Norway and Denmark [11,13,53,54]. For clinical managers and digital health leaders, the variations we found have implications for standardization and flexibility, especially variations between units in their volume of use of digital consultations. To ensure policies, laws, and guidelines are followed by primary care unit staff, especially about equity and quality of care, evidence will be needed to decide what needs to be standardized and monitored within one unit and across all units [29]. Standardization needs to be balanced with flexibility, if units and staff are to decide what is appropriate for their patient groups and for individual patients. Units know their populations and how well the CDCS works for them: their use of its CDCS may be low but correct for their populations. The same applies to individual clinicians if they have training to use the system.

Our study highlights critical questions that practitioners and researchers need to ask when considering any studies or guidance about a digital health technology and seeking to integrate digital and physical care. The most important are about the technology, implementation, and context, and how dynamic all three are:

What specifically is the CDCS technology and was it changed during the study? There are many types of systems for connecting patients and providers with many different characteristics that are important, such as which functions they cover (eg linking to other data systems such as an EHR), their ease of use by provider or patient, and their security. Evidence about one type of CDCS may not apply to another system, and it needs some knowledge and judgement to decide how similar or different the CDCS is to a system used by one service now and as planned. Also, informatics staff responsible for CDCS operations frequently make changes to the CDCDs and these may have been made during the study and not fully known or reported and may affect the data collected at different times. In our study, asking subjects about the last 18 months of the pandemic meant that they may have reported experiences with the CDCS before some changes were made.

### What is the extent of the implementation of the CDCS, and was the implementation strategy changed during the study?

Our findings relate to one CDCS in a health system, with limited training for clinical and management personnel about the CDCS. Later in the pandemic, more on-line education and guidance was available to them, but there was no formal implementation strategy and support to enable them to take up use of the CDCS in their everyday practice. In addition, the healthcare organization had achieved significant management decentralization and management flexibility for unit managers over a period of 14 years [24,32,33]. There may be significant differences to other primary care service in the competencies to use the CDCS and flexibility afforded to unit managers in their take up of digital services. These points may apply to studies of other CDCSs but may not be clear in a study report and may mislead readers thinking that the study findings apply to their service.

### What is the context for the CDCS and does this change?

A variety of other factors affect the use of CDCSs by clinicians and unit mangers that emanate at the unit, wider healthcare organization, and regional and national levels. These include regulations about data use and security, professional ethics and guidance, financial reimbursement and grants, market competition for patients, staff shortages, competencies and recruitment procedures, and availability of information specialist staff support. In our study the higher-level regulations were all stable, apart from one which changed to require on-line only clinical services to provide physical units in Stockholm if they were to be reimbursed by the public system. At least one of these, or other factors, will change, and can substantially affect the operation and implementation of the CDCS, but changes may not be reported in a particular study. These factors need to be understood by clinicians and unit mangers considering acting on findings from a study of a similar CDCS but carried out in a different setting.

### Adding knowledge to the NASSS framework

Overall, we found NASSS to be very helpful for analysing our data but we see an opportunity to offer our observations for its further development. The NASSS framework proved useful for capturing important aspects for introducing a new technology into a large health care provider organisation and its units [24]. Even though the developers of the NASSS give quite detailed guidance for classifying data into the seven domains, we found it necessary to use two strategies to make an adequate classification [55]. The first was to consider how others had interpreted the seven domains of the NASSS. [21,25,55–58]. The second was when focus group members statements could be classified in more than one domain, for example, “the app is not very user friendly for some patients”. Should this be classified as an issue of the technology (domain two), or the value proposition (domain three), or the adopter system (domain four), or possibly the wider context (domain six) or classified in all or some of these? The strategy of the research team of three (the authors) was to discuss and agree how to classify the statements made by focus groups members and the codes.

**Figure 1.**
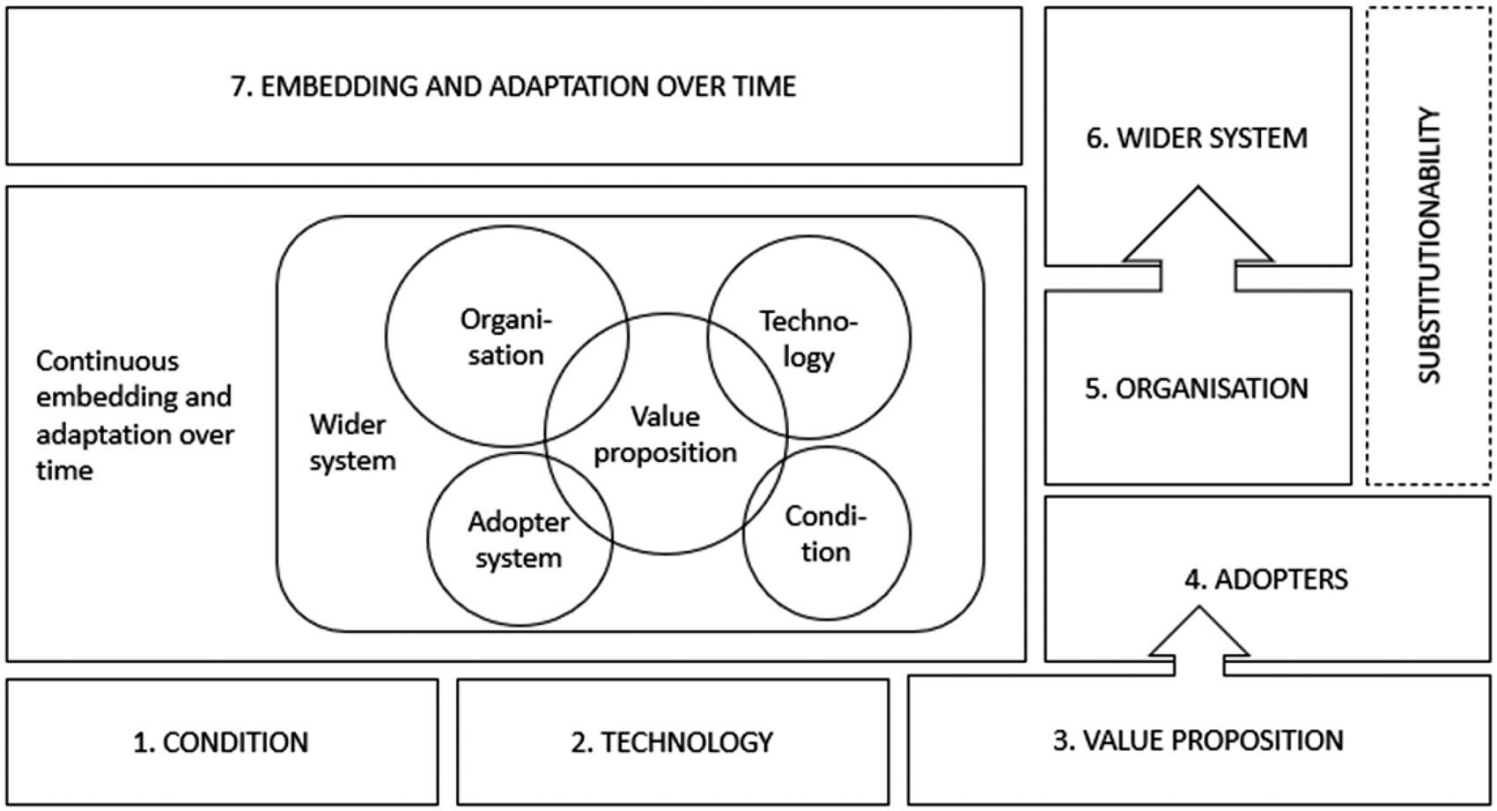
Proposed further development of the NASSS framework.

We also noted challenges with classification in relation to some domains and suggest there is scope to develop NASSS further to address these issues. One way forward could be to develop versions of NASSS for different types of digital health technologies, such as, in our case, a development of an existing technology and not an original innovation. Based on our experiences using NASSS for this study and setting, our current vision regarding development of the model is described as pictured in Figure 1.

In domain three (value proposition), we suggest that the definition could be more specified on the supply-side. We found in our study that personnel value the technology because it makes their work more flexible and enables them to work from home. It is not only a “role changer” as defined in domain four (adopter system), but also a strong driver as a value proposition. Also, how easy it is for competitors to enter the market with their version is placed under domain two (technology) but, in our case, belongs between domain five (organisation) and six (wider system) as it is a development of an existing technological innovation.

### Limitations

This is a study set in a specific setting, studying the implementation of an already existing technology which was scaled-up due to the emerging events of the COVID-19 pandemic. Our findings are a product of these contextual factors, in addition to the organizational and cultural factors that normally influence an implementation process. We strived to describe these circumstances in such a way that transferability may be possible to settings in a similar contextual situation. We chose to interview the different clinician-managers due to their given role of being drivers for adoption of the new technology. However, the practitioners are the everyday users of the new technology, and this study does not directly capture their experiences, which is also of interest. This could be a perspective for further studies.

We recognize the inherent recall bias of asking interviewees to reflect on development work after a period of 18 months. In this case, we believe that the recall bias may be lessened by utilizing group interviews, since this method may help the group recall events together. However, a limitation of the study are the differences between the twelve focus groups. First, the number of participants varied from two in one group to sixteen in another. In a focus group of sixteen there is less time for each person to give their perceptions and also individuals may hesitate to give views that conflict with what they perceive as a group consensus compared with a smaller group. In a small group individuals have more time to explain why their perceptions are different and may feel they are being less disruptive to the group. Although twelve groups with 99 clinician-managers are likely to have elicited a comprehensive range of perceptions, these differences could have influenced both whether certain views were presented as well as the researchers’ assessments of the importance or weighting to give to the perceptions documented.

## Conclusions and implications

In line with the NASSS framework, our findings suggest that, although staff are positive toward using a digital technology system for remote care, factors concerning other areas of the health care system may play a key role in how well it can be implemented to provide and further develop both digital and physical care.

Participants of the focus groups reported a rapid adoption of the digital system and video consults, a generally positive experience but also some weaknesses of the system. Different to other studies, we found a continual high use of the system by units that had adopted it, and a reported intention to sustain their use of the system. In addition, they want to actively contribute to developing and better integrate AO into their work processes as part of the digiphysical-care strategy. We keep researching how its implementation and continued use develops.

Many factors could explain the rapid but variable adoption. We speculate that *one* was the 14-year management decentralization programme in the primary and community health care organization. Our previous studies have revealed that this has developed the capability of unit managers to provide flexible services suited to local needs. This factor will be further scrutinised in upcoming research.
